# The effect of recombinant human soluble thrombomodulin on renal function and mortality in septic disseminated intravascular coagulation patients with acute kidney injury: a retrospective study

**DOI:** 10.1186/s40560-020-00512-w

**Published:** 2020-12-11

**Authors:** Masayuki Akatsuka, Yoshiki Masuda, Hiroomi Tatsumi, Tomoko Sonoda

**Affiliations:** 1 Department of Intensive Care Medicine, Sapporo Medical University School of Medicine, South 1, West 16, Chuo-ku, Sapporo, Hokkaido 060-8543 Japan; 2 Department of Public Health, Sapporo Medical University School of Medicine, Sapporo, Japan

**Keywords:** Recombinant human soluble thrombomodulin, Sepsis, Acute kidney injury, Disseminated intravascular coagulation

## Abstract

**Background:**

Clinical evidence showing the effectiveness of recombinant human soluble thrombomodulin (rhTM) for treating sepsis-induced disseminated intravascular coagulation (DIC) and organ dysfunction (particularly renal injury) is limited because of differences in the inclusion criteria and disease severity among patients. This study aimed to assess the association between rhTM and outcomes in septic DIC patients with acute kidney injury (AKI).

**Methods:**

This retrospective observational study analyzed the data of patients who were admitted to the intensive care unit (ICU) of a single center between January 2012 and December 2018, and diagnosed with sepsis-induced DIC and AKI. Data were extracted as follows: patients’ characteristics; DIC score, as calculated by the Japanese Association for Acute Medicine and the International Society of Thrombosis and Hemostasis criteria; serum creatinine levels; and ICU and 28-day mortality rates. The primary outcome was the dependence on renal replacement therapy (RRT) at ICU discharge. The propensity score (PS) was calculated using the following variables: age, sex, septic shock at admission, DIC score, and KDIGO classification. Subsequently, logistic regression analysis was performed using the PS to evaluate the outcome.

**Results:**

In total, 97 patients were included in this study. Of these, 52 (53.6%) patients had received rhTM. The dependence on RRT at ICU discharge was significantly lower in the rhTM than in the non-rhTM group (odds ratio [OR], 0.43; 95% confidence interval [CI], 0.19–0.97; *P =* 0.043). The serum creatinine levels at ICU discharge (OR, 0.31; 95% CI, 0.13–0.72; *P* = 0.007) and hospital discharge (OR, 0.25; 95% CI, 0.11–0.60; *P* = 0.002, respectively), and the 28-day mortality rate (OR, 0.40; 95% CI, 0.17–0.93; *P* = 0.033) were significantly lower in the rhTM than in the non-rhTM group. Moreover, the Kaplan–Meier survival curve revealed significantly lower mortality rates in the rhTM than in the non-rhTM group (*P* = 0.009). No significant differences in the DIC score and AKI severity were observed between the groups.

**Conclusions:**

Among sepsis-induced DIC patients with AKI, rhTM administration was associated with lower dependence on RRT at ICU discharge, improvement in renal function, and lower 28-day mortality rate.

## Background

Despite advances in modern medicine, sepsis and septic shock remain the leading causes of death in critically ill patients [1–3]. In 2016, the definition of sepsis was changed from “infection-induced systemic inflammatory response syndrome” to “infection-induced organ failure.” Sepsis-induced organ failure is one of the leading causes of sepsis-associated mortality. Large amounts of inflammatory mediators are produced during sepsis, thus, inducing endothelial cell perturbation and resulting in coagulopathy and disseminated intravascular coagulation (DIC). Septic coagulopathy includes activation of coagulation, inhibition of fibrinolysis, and depletion of coagulation hemostatic factors, resulting in inadequate fibrin deposition in the microcirculation and, subsequently, in organ failure [4–6]. Endothelial perturbation was reported to be responsible for the sepsis-induced organ failure development [7].

Respiratory failure, coagulopathy, and acute kidney injury (AKI) are among the most common sepsis-related organ failures [8]. AKI is reported to occur in 30–40% of patients with sepsis and septic shock [9–12]. In addition, a recent study reported that coagulopathy is associated with the development of AKI [4]. In combination, coagulopathy and AKI are responsible for more than 70% of mortality incidences in patients who developed organ failure [8]. Therefore, anti-inflammatory and anticoagulant therapeutic approaches may play a crucial role in preventing the development of multiple organ failure, following septic coagulopathy.

Physiologically derived anticoagulant proteins, such as thrombomodulin (TM) and antithrombin, have been used for the treatment of septic coagulopathy. TM is a thrombin-binding anticoagulant cofactor expressed on the surface of endothelial cells. Moreover, it plays an important role in the regulation of intravascular coagulation [13]. Recent studies have shown that the recombinant human-soluble TM (rhTM) inhibits the production of inflammatory cytokines and binds to high-mobility group box-1 protein, also known as one of the damage-associated molecular patterns (DAMPs) [14, 15]. Some clinical studies have demonstrated that rhTM administration improves the mortality rates in septic DIC patients; however, the impact of rhTM on organ failure has not been investigated thoroughly. In a previous study, we demonstrated the efficacy of rhTM on renal injury in an experimental model of rats with induced sepsis [16]. Thus, this study aimed to assess the association between rhTM and the obtained outcomes after performing this treatment in septic DIC patients with AKI.

## Methods

### Study design and patient selection

This retrospective observational study was performed in the intensive care unit (ICU) of the Sapporo Medical University Hospital (Sapporo, Hokkaido, Japan) between January 2012 and December 2018. Septic DIC patients with AKI were enrolled in this study. Patients were diagnosed with sepsis and DIC at ICU admission according to the SEPSIS-3 definition [17] and the DIC criteria established by the Japanese Association for Acute Medicine (JAAM) [18], respectively. AKI was confirmed using the Kidney Disease Improving Global Outcomes (KDIGO) criteria [19] at ICU admission. Patients who required hemodialysis before ICU admission and those aged < 18 years were excluded.

Patients were classified into two groups: those who were administered rhTM and those were not. Moreover, the patients who did not receive RRT and received rhTM prior to RRT were excluded to perform statistical analysis using propensity score to adjust the patients’ characteristics. In each case, the decision regarding the administration of anticoagulants and selection of drugs for DIC was taken after a discussion between the attending physician and the intensivist. Administration of rhTM was started within 24 h of septic DIC diagnosis. The dosage of rhTM was 0.06 mg/kg/day, and it was administered for 30 min once a day for 6 days. The other anticoagulant drugs used for DIC were unfractionated heparin and plasma-derived antithrombin drugs.

The study design and protocol were approved by the Institutional Review Board of Sapporo Medical University (approval number: 312-54, UMIN000037245), and the requirement for obtaining informed consent was waived because of the retrospective design of the study.

### Data collection

The following data were collected from the electronic health records of the patients: age, sex, body mass index, Acute Physiologic and Chronic Health Evaluation II (APACHE II) score, Sequential Organ Failure Assessment score on ICU admission, infectious foci, presence of septic shock at ICU admission, DIC score, as defined by the JAAM [18] and the International Society of Thrombosis and Haemostasis criteria [20], anticoagulant administration, steroid use, KDIGO classification, timing of RRT from ICU admission, duration of ICU stay, number of days on ventilator support, dependence on RRT at ICU discharge, number of days of RRT, serum creatinine levels at ICU admission, ICU and hospital discharge for survivors, and ICU and 28-day mortality rates.

### Measurement of the outcomes

The primary outcome was the dependence on RRT at ICU discharge. The secondary outcomes were the serum creatinine levels at ICU admission, ICU discharge, and hospital discharge; number of RRT-free, ICU-free, and ventilator-free days at the 28-day time-point; and the ICU and 28-day mortality rates.

### Statistical analysis

The categorical variables are expressed as numbers and percentages. The continuous variables are expressed as means and standard deviations or as medians and interquartile ranges (IQR), as deemed appropriate. The categorical and continuous variables were analyzed using the chi-square and Mann–Whitney *U* tests, respectively. The propensity score (PS) approach was performed to address the selection bias inherent in a retrospective observational study. The PS was calculated by predicted probabilities for rhTM in logistic regression analysis, using variables (i.e., age, sex, shock, DIC score, and KDIGO classification) to adjust for the confounding factors. Subsequently, logistic regression analysis using the PS as the adjusted variable was performed to determine the risk estimate for the association between rhTM treatment and the primary or secondary outcomes.

The proportion of patients who died within 28 days was assessed, and the corresponding survival probabilities were plotted using the Kaplan–Meier method. The survival curves obtained using the Kaplan–Meier analysis were compared using the log-rank test. Statistical analyses were performed using IBM SPSS Statistics version 27 (IBM, Armonk, NY, USA). A *P* value < 0.05 was considered statistically significant.

## Results

**Fig. 1:**
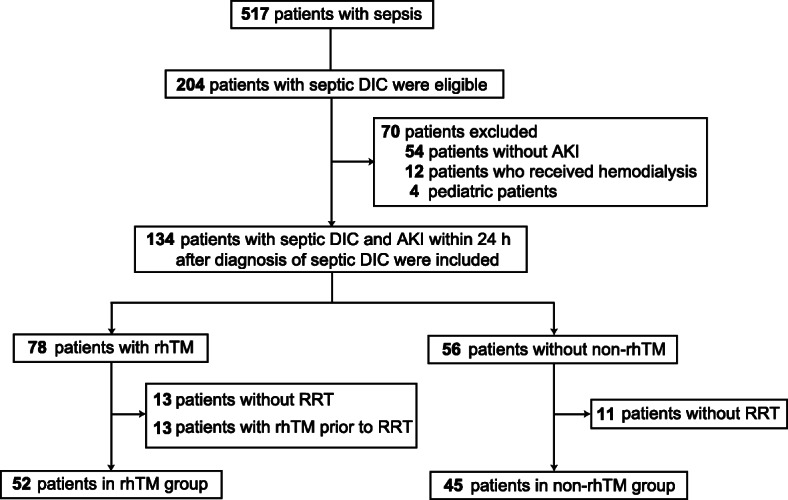
Flow diagram of this study. AKI, acute kidney injury; DIC, disseminated intravascular coagulation; rhTM, recombinant human soluble thrombomodulin; RRT, renal replacement therapy

In total, 97 eligible patients were identified during the study period between January 2012 and December 2018. The rhTM and non-rhTM groups comprised 52 (53.6%) and 45 patients (46.4%), respectively. A flow diagram showing patient enrollment is presented in Fig. 1.

### Baseline characteristics

The baseline characteristics of the patients enrolled in the study were well-balanced between the two groups (Table 1). No significant differences were observed in the baseline characteristics between the rhTM and non-rhTM groups. The median (IQR) timing of rhTM administered was 10.5 (IQR, 3.5–20.3) h.

**Table 1:** Characteristics of the patients

	rhTM group (*n* = 52)	Non-rhTM group (*n* = 45)	*P* value
Age (years)	66 (54–74)	65 (51–73)	0.90
Male, *n* (%)	35 (67.3)	35 (77.8)	0.27
Body mass index	22.1 (19.4–24.0)	23.1 (20.6–25.9)	0.23
APACHE II	25 (22–27)	25 (21–30)	0.33
SOFA score at ICU admission	9 (6–10)	9 (7–11)	0.34
Focus of infection			
Abdomen	22	15	
Lung	19	16	
Urogenital tract	3	3	
Skin and soft tissue	3	2	
CRBSI	2	1	
Miscellaneous	3	8	
Septic shock, *n* (%)	37 (71.2)	33 (73.3)	0.83
DIC score			
JAAM DIC	6 (5–7)	6 (5–7)	0.52
ISTH overt DIC	6 (5–6)	6 (4–6)	0.76
Anticoagulants, *n* (%)			
rhTM	52 (100)	-	
Heparin	-	18 (40)	
Antithrombin	30 (57.7)	18 (40)	0.10
Others	-	8 (17.8)	
Steroids, *n* (%)	32 (61.5)	18 (40)	0.16
KDIGO classification			0.57
Stage 1	16	17	
Stage 2	19	12	
Stage 3	17	16	
Timing of RRT from ICU admission (h)	3.8 (2.0–7.9)	4.0 (3.0–5.8)	0.67

Data are presented as medians (IQR). The body mass index is the weight in kilograms divided by the square of the height in meters. The SOFA score ranges from 0 to 24, with higher scores indicating more severe organ failure. The APACHE II score ranges from 0 to 71, with higher scores indicating more severe disease and a higher risk of death. Acute kidney injury was defined according to the KDIGO classification. Stage 3 of the KDIGO classification is defined as a serum creatinine level three times the baseline level or an increase of ≥ 4.0 mg per deciliter, oliguria (urine output < 0.3 mL per kilogram of body weight per hour for ≥ 24 h), or anuria for ≥ 2 h. The baseline serum creatinine levels were determined using values measured at 12 months preceding the ICU stay or were estimated

*ICU* intensive care unit, *IQR* interquartile range, *CRBSI* catheter-related bloodstream infections, *DIC* disseminated intravascular coagulation, *JAAM* Japanese Association for Acute Medicine, *ISTH* International Society on Thrombosis and Haemostasis, *RRT* renal replacement therapy, *rhTM* recombinant human soluble thrombomodulin, *SOFA* Sequential Organ Failure Assessment, *APACHE II* Acute Physiology and Chronic High Evaluation II, *KDIGO* Kidney Disease Improving Global Outcomes

The outcomes of performing the logistic regression analysis using the PS as the adjusted variable are presented in Table 2.

**Table 2:** Odds ratios regarding the primary and secondary outcomes in the rhTM and non-rhTM groups

	rhTM group (*n* = 52)	Non-rhTM group (*n* = 45)	Regression coefficient	OR (95% CI)	*P* value
**Primary outcome**					
Dependence on RRT at ICU discharge—no. (%)	21 (40.4)	28 (62.2)	− 0.86	0.43 (0.19–0.97)	0.043
**Secondary outcomes**					
Serum creatinine level (mg/dL)					
at ICU admission	2.00 (1.19–2.87)	2.30 (1.50–3.14)	− 0.39	0.67 (0.30–1.52)	0.34
at ICU discharge	1.22 (0.77–1.75)	2.23 (1.09–2.94)	− 1.18	0.31 (0.13–0.72)	0.007
at hospital discharge	1.00 (0.74–1.62)	1.98 (1.04–2.94)	− 1.37	0.25 (0.11–0.60)	0.002
RRT-free days for 28 days	15 (0–21)	0 (0–21)	1.06	2.89 (1.24–6.74)	0.014
ICU-free days for 28 days	13 (0–20)	0 (0–17)	0.90	2.47 (1.07–5.67)	0.033
Ventilator-free days for 28 days	20 (0–23)	0 (0–24)	1.20	3.31 (1.41–7.75)	0.006
Mortality					
ICU—no. (%)	18 (34.6)	22 (48.9)	− 0.59	0.56 (0.24–1.28)	0.17
28 days—no. (%)	15 (28.8)	23 (51.0)	− 0.92	0.40 (0.17–0.93)	0.033

Data are presented as medians (IQR). The ICU-free and mechanical ventilation-free days were calculated according to the number of days in which the patient was alive and did not receive the specified therapy during the first 28 days after enrollment; patients who died were assigned as having 0 free days. The number of RRT-free days was calculated according to the number of days in which the patient did not receive RRT during the first 28 days after enrollment

*RRT* renal replacement therapy, *ICU* intensive care unit, *IQR* interquartile range, *OR* odds ratio, *95% CI* 95% confidence interval, *rhTM* recombinant human soluble thrombomodulin

### Primary outcome

Dependence on RRT at ICU discharge was significantly lower in the rhTM than in the non-rhTM group (odds ratio [OR], 0.43; 95% confidence interval [CI], 0.19–0.97; *P* = 0.043; Table 2).

### Secondary outcomes

No significant differences were observed in the serum creatinine level at ICU admission between the rhTM and non-rhTM groups. However, the serum creatinine levels at ICU discharge and hospital discharge were significantly lower in the rhTM than in the non-rhTM group (OR, 0.31; 95% CI, 0.13–0.72; *P* = 0.007 and OR, 0.25; 95% CI, 0.11–0.60; *P* = 0.002, respectively). The number of RRT-free days was significantly greater in the rhTM than in the non-rhTM group (OR, 2.89; 95% CI, 1.24–6.74; *P* = 0.014). Moreover, the number of ICU-free and ventilator-free days differed significantly between the two groups (OR, 2.47; 95% CI, 1.07–5.67; *P* = 0.033 and OR, 3.31; 95% CI, 1.41–7.75; *P* = 0.006, respectively).

**Fig. 2:**
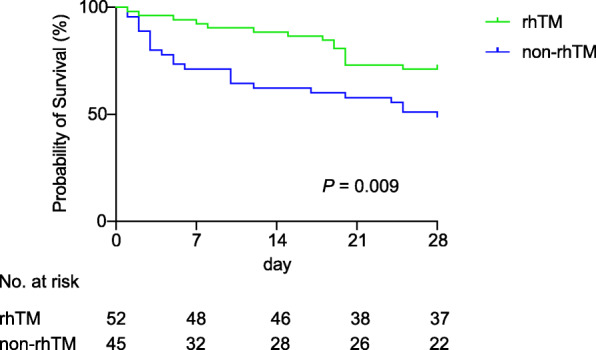
Kaplan–Meier survival curves for septic DIC patients with AKI assigned to the rhTM and non-rhTM groups. The green and blue lines represent patients in the rhTM and non-rhTM groups, respectively. The rhTM treatment was associated with a significantly higher survival rate (*P* = 0.009). AKI, acute kidney injury; rhTM, recombinant human soluble thrombomodulin

The ICU mortality rate was not significantly different between the patients who received rhTM and those who did not (OR, 0.56; 95% CI, 0.24–1.28; *P* = 0.17). The 28-day mortality rate was significantly lower in the rhTM (28.8%) than in the non-rhTM group (51.0%) (OR, 0.40; 95% CI, 0.17–0.93; *P* = 0.033). Similarly, the 28-day mortality was significantly lower in the rhTM group (*P* = 0.009), as shown in the Kaplan–Meier survival curve (Fig. 2).

## Discussion

The results of our study showed that the rate of dependence on RRT at ICU discharge and the serum creatinine levels at ICU and hospital discharge decreased in septic DIC patients with AKI who received rhTM therapy. Moreover, rhTM therapy contributed to lower mortality rates at 28 days among septic DIC patients with AKI than among those who did not receive rhTM therapy. These results indicated that rhTM can potentially protect the renal function of patients with septic coagulopathy. Furthermore, this study suggested that rhTM administration in septic DIC patients with AKI may improve the overall prognosis.

Sepsis is associated with a high incidence of AKI complications, while AKI occurs in approximately 22–53% of septic patients [21–24]. In addition, sepsis complicated with AKI is associated with an increase in mortality rates ranging from 38 to 41% [21, 24]. Moreover, the prognosis of renal function in septic patients with AKI is comparatively poor, and approximately 20–50% of patients develop chronic kidney diseases [25]. Therefore, it is important to develop strategies to prevent the onset of septic AKI. Several methods have been reported for the prevention of renal injury during sepsis treatment as follows: identifying patients at risk of developing AKI, early treatment intervention for sepsis, refraining from the use of nephrotoxic drugs, and optimizing cardiac output and mean arterial pressure to maintain perfusion pressure of the kidney [26]. However, the management of septic AKI after its onset has not been adequately investigated. Therefore, it is important to develop treatment plans to improve sepsis-induced renal dysfunction and prognosis.

The pathophysiology of septic AKI includes many factors, such as inflammation and dysregulated immune response, microangiopathy, and induction of cell disorders. An excessive inflammatory response is mainly responsible for sepsis pathology, while inflammatory mediators and DAMPs could be released in the process, which ultimately lead to organ dysfunction. Therefore, it is important to inhibit these mediators, including DAMPs, to protect and improve sepsis-induced renal dysfunction.

rhTM consists of three domains of the TM extracellular region expressed on vascular endothelial cells. It comprises the extracellular domain of TM. The epidermal growth factor-like domain of rhTM is known to bind thrombin, which could possibly result in anticoagulant action. The lectin-like domain of rhTM plays a crucial role in binding some kinds of DAMPs, such as HMGB-1 [27]. In addition, rhTM exerts its anti-inflammatory function via activated protein c and the thrombin activatable fibrinolysis inhibitor pathway [28]. Therefore, rhTM has presented promising antithrombotic and anti-inflammatory activities [29]. Furthermore, in the experimental model of septic rats, rhTM administration improved sepsis-induced kidney injury by inhibiting the release of intracellular histone H3 (a known DAMP) into the extracellular space [16]. These mechanisms of rhTM may help improve the pathophysiology of septic AKI.

DIC is characterized by the activation of the coagulation system and thrombotic obstruction of microcirculation, resulting in organ ischemia and dysfunction. Sepsis is the most common cause of DIC, with 20–40% of septic patients presenting DIC [30, 31]. In addition, DIC is an independent and relatively reliable predictor of organ dysfunction and mortality in septic patients [32–34]. Therefore, anticoagulant therapy is one of the important treatment strategies for improving the pathophysiology of disseminated thrombus formation in systemic microvessels in septic DIC patients. A previous study reported decreased mortality and improved survival rates upon rhTM administration in such patients, demonstrating its clinical efficacy in improving prognosis [35]. Conversely, another recent study (SCARLET study) showed that rhTM administration for sepsis-associated coagulopathy was not associated with 28-day mortality [36]. However, the inclusion criteria of SCARLET study differed from those of our study, particularly regarding the inclusion of patients who were not diagnosed with DIC.

The reported mortality rate of septic patients complicated with coagulopathy and other organ failures was 50–70% [8], verifying coagulopathy as an important factor influencing prognosis. Especially, the mortality rate of patients who developed coagulopathy and renal failure was reported to be approximately 67% [8]. Hence, appropriate treatment strategies are required for coagulopathy and renal dysfunction in septic patients. rhTM has been indicated to elicit an anticoagulant and a renal-protective effect [16]. This finding suggested that rhTM could be an effective drug for the treatment of septic DIC patients with AKI. To the best of our knowledge, this is the first study demonstrating the renal-protective and prognosis-improving effects of rhTM in septic DIC patients complicated with AKI, suggesting a novel role for this recombinant drug. In the future, a further prospective study will be required to clarify the efficacy of rhTM in such patients.

Our findings have several potential clinical implications. First, decreased dependence on RRT could improve the quality of life of patients after ICU discharge. Second, a therapeutic strategy in patients with multiple organ failure, including coagulopathy, is hard to be implemented, as such patients are known to have higher mortality [8]. Therefore, administration of rhTM against coagulopathy may be a potential therapeutic option for patients with multiple organ failure, especially those with septic DIC and AKI.

Although our study results highlighted several important findings, there were several limitations that should be acknowledged. First, as it was a retrospective observational study rather than a randomized controlled trial, it may be difficult to generalize our results. Second, the sample size was relatively small, as it was a single-center study. Therefore, future multicenter studies including more septic DIC patients with AKI are warranted. Third, our results did not consider differences in the use of nephrotoxic drugs (such as antibiotics). Finally, as rhTM therapy is only performed in Japan, it may not be common in other countries.

## Conclusions

This study showed a significant difference between septic DIC patients with AKI who received rhTM therapy and those who did not concerning the dependence on RRT at ICU discharge and the serum creatinine levels. Furthermore, rhTM administration was associated with a decreased 28-day mortality rate. Therefore, rhTM therapy may be a potential therapeutic option for septic DIC patients with AKI, and prospective clinical trials should be initiated to evaluate the efficacy of rhTM therapy in this patient population.

## Data Availability

The dataset generated and analyzed in this study is not publicly available because of patient-related confidentiality but is available from the corresponding author upon reasonable request.

## Abbreviations

APACHE II: Acute Physiologic and Chronic Health Evaluation II score
AKI: Acute kidney injury
BMI: Body mass index
RRT: Renal replacement therapy
DAMPs: Damage-associated molecular patterns
DIC: Disseminated intravascular coagulation
ICU: Intensive care unit
JAAM: Japanese Association for Acute Medicine
KDIGO: Kidney Disease Improving Global Outcomes
rhTM: Recombinant human soluble thrombomodulin
